# Responsible data science is a responsibility for all

**DOI:** 10.1016/j.patter.2022.100607

**Published:** 2022-10-14

**Authors:** Isabelle Fest, Maranke Wieringa, Ben Wagner

**Affiliations:** 1 Utrecht School of Governance, Utrecht University, Utrecht 3511ZC, the Netherlands; 2 Governing the Digital Society, Utrecht University, Utrecht 3512 BR, the Netherlands; 3 Parell Group, Arnhem 6824 BJ, the Netherlands; 4 Department of Technology, Policy and Management, TU Delft, Delft, the Netherlands; 5 Faculty of Creative Futures, Inholland University of Applied Science, The Hague, the Netherlands

## Abstract

In a collaboration between TU Delft and Utrecht University, the authors recently wrote a qualitative study on the gap between regulatory (ethical and legal) frameworks and the daily practices of data professionals. This People of Data highlights the importance of collaboration in the success of interdisciplinary research and the responsibility of data scientists in safeguarding the public and ethical values.

## Main text

### What would you like to share about your background (personal and/or professional)?

**Isabelle Fest:** I am a PhD researcher at Utrecht University, and part of the ALGOPOL project on algorithmic policing. Prior to my PhD I worked as a policy researcher/adviser at the court of audit of the Dutch provinces North-Brabant and Limburg. I started my academic career studying electrical engineering, but I spent more time being social than I did on my studies and ended up switching within the year. In a complete 180, I obtained my bachelor’s degree in Dutch language and culture in 2015 and continued with a (joint) master’s degree in Euroculture, which basically entails a study of European politics, culture, and identity mixed with a bit of international relations. I studied at Georg-August University Göttingen and Jagiellonian University Kraków and was also able to spend a research semester at Osaka University. My interdisciplinary career really reflects my personality, as I find almost everything interesting and am a naturally curious person.

**Maranke Wieringa:** I am an external PhD candidate at Utrecht University and work as a consultant on topics such as data-driven work in government, algorithmic use by the government, and the likes. My background is a tad unconventional as I always oscillated between practice and theory. I briefly studied cabaret, then cultural and social development, after which I eventually settled on cultural studies. After my BA, I completed an rMA in media studies and worked as a researcher at Utrecht Data School and a lecturer at Utrecht University and University of Applied Sciences Utrecht. In 2018, I started my PhD project but decided to leave the university as an employee in 2022 due to the institutional ableism I experienced as a disabled scholar. I now work as a consultant and continue my research as an external PhD.

**Ben Wagner:** I am an assistant professor at TU Delft and professor of media, technology and society at Inholland. I’ve spent many years working in tech but did my PhD in social and political science at European University Institute in Florence. I enjoy researching the intersection of technology and society, typically outside of the “usual” countries and contexts where it is studied.

### What motivated you to become a researcher? Is there anyone/anything that helped guide you on your path?

**IF:** When I did a research internship during my bachelor’s, I was completely disillusioned with doing research. I found the whole experience so stressful that I swore I would never do research again. But during my master’s, I experienced creative freedom in my research topics and methods. Research became fun, and I enjoyed thinking about it so much! Looking back, I should have known. The same aspect draws me to my hobbies, e.g., making cosplay costumes. What makes cosplay fun is the hours upon hours I spend mulling over designs in my mind, thinking what would look most realistic, what kinds of adhesives stick to what materials best and how one can actually move in a costume. It is the puzzles and creative thinking that drove me to cosplay, and when I found that research is not so different at all, I started enjoying it.

**MW:** The relationship between myself and academia has always been rather strenuous. Honestly, it’s an on-again, off-again relationship. I love both the deeply theoretical bits of being an academic, and the “getting your hands dirty” bits of working in practice. For me, ultimately, theory is only interesting when I can use it in practice. This is not necessarily something that is appreciated in a humanities faculty. It was part of the reason why I became very disillusioned during my, rather theoretical, research master program. That is, until I found Utrecht Data School, where I could do exactly this kind of practical yet challenging research. I was elated! I eventually decided to pursue a PhD on a topic and research design of my own choosing. I eventually had to give up on a full-time PhD pursuit due to institutional ableism. Now I happily combine my PhD research and my work as a consultant.

### What drew you to your current team and topic?

**IF:** After finishing my studies, I was already convinced that I would like to do a PhD at some point. Knowing how tough PhD life can be, I felt like I needed to find a project where I could stay motivated for the full duration of the research. In my case, the project needed to be interdisciplinary—ideally with some technology aspect—and offer me a lot of freedom and autonomy to craft my own PhD. I found both these things in ALGOPOL, and that is what drew me to apply. It was the inspiring supervisors who can be considered real experts in their respective fields, and the informal and direct way of communicating we had established right from the start that made me jump for joy when I was offered the position.

### How did this project you wrote about come to be?

**IF:** Luck, good timing, and friendship! I consider Maranke one of my close personal friends. We have an open, non-competitive relationship, and we often share ideas and interesting findings with one another. We always thought it would be fun to work on an article together, if the opportunity would arise. At one point we discovered similar patterns in both our research data, despite municipalities and police being very different environments.

As luck would have it, this realization converged with a meeting between my co-authors to catch up and an opportunity to write for *Patterns*. It was a matter of speaking with the right people at the right time. Maranke introduced me to Ben, and we had an instant understanding, which made for smooth and enjoyable collaboration.

### Who were the driving forces behind the project?

**MW:** I’d say Isabelle, Ben, and joy. This really was a fun project that also made it really enjoyable to pick up even though my life as an alt-ac consultant is more hectic and it places more constraints on my time management.

### Was there a particular result that surprised you, or did you have a eureka moment? How did you react?

**IF:** At one point when we were working with our framework^1^ to analyze our data, we got really stuck. We found points of the framework to be overlapping and felt we were constantly repeating ourselves. At the same time, for some of the things we wanted to say, we just did not quite know where to put it. Nothing really seemed to fit. I got really frustrated, until we had our eureka moment where everything got very meta for a while: this was exactly what was wrong with frameworks. They concern lofty principles and are very far from the practical reality they are supposed to regulate. By working with a framework, we were proving our own point.

### What is the definition of data science in your opinion? What is a data scientist? Do you self-identify as one?

**IF:** The concept data science, in my opinion, does not exist in a vacuum with a predetermined meaning. When approached from different perspectives, it gets different meanings. A user or a manager, for example, will have very different ideas about a technology or the science behind it than a data scientist. Such ideas and conceptions have an impact on their behavior and, therefore, impact how data science functions in our world. It is exactly this diversity (or “multiplicity,” see e.g., Mol^2^) of meanings that is relevant to my research. Giving a singular definition of data science would be counterproductive and might even be hypocritical. It is probably not surprising I do not self-identify as data scientist.

**MW:** Data science seems to mean so many different things to different people. I could give you a textbook definition, but that doesn’t seem to cover it. As Isabelle notes, this multiplicity makes it difficult to pin it down for me. I do not identify as a data scientist, but I do identify as a data analyst, amongst a bunch of other identities. The difference, based on my gut feeling? Less intimidating statistics.

### What barriers have you faced in pursuing data science as a career?

**MW:** So, I don’t identify as a data scientist, but I always wanted to learn more about data science and particularly statistics. I have tried 3–4 times but have yet to find a lecturer who can explain statistics in a way that “fits” my neurodivergent brain. My brain is wired so that I can only understand A Small Aspect of A Big Thing if I understand the Big Thing itself. But that’s not how statistics (or many other kinds of science) are typically taught. We typically break a Big Thing down into Small Aspects and then eventually glue them together for our students and go: “Yeah, so this was part of A Big Thing all along, surprise!” I sometimes compare this to learning how to crochet. My mother has tried teaching me how to crochet three times by teaching me how to do particular stitch. I had to perfect that one before moving on to the next stitch. But until I had a neurodivergent friend explain to me the “anatomy” of crocheting, how to read the pattern, and so forth, I was just meaninglessly attempting to practice my stitches without any idea how I would fit them in a crochet piece. Similarly with statistics, I’ve never had a teacher explain the “anatomy” of statistics before telling me which SPSS buttons to push for an ANOVA or what have you. And so it unfortunately just never “clicked” for me.

### What is the role of data science in your domain/field? What advancements do you expect in data science in this field over the next 2–3 years?

**IF:** Public institutions are highly interested in data science, and there is an increasing amount of experimentation in government with data science practices and related technologies. I try not to dabble too much in fortune telling, but this seems to be a trend that is likely to stay with us for some time. Government organizations believe there is a lot to gain from data science, especially in terms of effectiveness, efficiency, and transparency, perhaps even sustainability in policymaking.

I am happy to recognize increasing awareness of public and ethical values and risks in such developments. Some values (e.g., privacy) are commonly considered at this point. I hope the less obvious values (e.g., autonomy, contestability, inclusion) will also become more visible. A second hope I foster is that values are considered beyond development, e.g., during actual use, evaluation, or implementation in the organization.

**BW:** I completely agree with what Isabelle said. We consider too much specific values and rights that are more frequently discussed like privacy or freedom of expression but less others that are not immediately evident. I would definitely want to add solidary, justice, and accountability to autonomy, contestability, and inclusion.

For the field more broadly, I would expect an increase of mainstreaming and specialization. It is increasingly normal to include data science skills in degree programs outside of typical data science specializations, which has the effect of mainstreaming some data science skills. At the same time, I see increased specialization in many areas, with data scientists becoming increasingly specialized in specific domains, such as accountability in security and policing.

### Which of the current trends in data science seem most interesting to you? In your opinion, what are the most pressing questions for the data science community?

**MW:** I am very happy to see a growing interest in algorithmic accountability. Such questions—knowing what our tools do, how they influence us, our work, and society—are among the most pressing questions of our ever-digitalizing society.

### Have you ever used your data science skills in your personal life? If yes, how?

**MW:** Yes! I have, for instance, used my data analysis skills to determine reasonable bids for real estate while my partner and myself were house-hunting.

### Why did you decide to publish in *Patterns*?

**IF:** I study data scientists and their work but rarely get the opportunity share my research outcomes with them, so I was really excited about *Patterns* as an up and coming data science journal. *Patterns* has shown to be very open to work of those disciplines—like mine—that might not traditionally be included in data science conversations.

### How did you celebrate your paper being accepted to *Patterns*?

**IF:** This was my first publication as part of my PhD dissertation, so quite a milestone. My celebration included overexcited messaging of friends and family that the paper was accepted. My partner and dog had to endure most of it, as I could not stop rambling on about it the rest of that day.

### What advice would you have given yourself at the start of the project? Is there anything you would have done differently?

**MW:** We actually gave ourselves some advice at the start of this collaboration. At the top of our document we wrote down in a big font: “Just have some fun!” and I think that is the best advice I can give anyone. Yes, of course scholarly or scientific work is serious business, but that doesn’t mean you can’t enjoy the company of fellow bright minds, have a laugh, and enjoy what you are doing. I personally find that when “it does not spark joy,” my writing style changes as well. It becomes flat, dull, and just drones on. Like, it will tick all of the academic boxes, but it just doesn’t have a soul.

### How did you come to collaborate?

**BW:** As an academic researcher at TU Delft working on accountable AI, I was a big fan of the FAccT (fairness, accountability, and transparency) work of Maranke Wieringa and wanted to get to know them better. I was really impressed by what they had published at what to me felt like quite an early stage in their academic career and thought it would be fun to chat and discuss ways to collaborate. Maranke introduced me to Isabelle, and we hit it off from there.

During the collaboration as an assistant professor at TU Delft, I was concerned that senior professors at Utrecht University would become very protective of their PhD candidates during the joint article writing process. I was pleasantly surprised how supportive their PhD supervisors were of the joint process. Both Maranke and Isabelle are amazing academics and great professionals, so even when nobody had time and work pressure was high, we were able to make progress on our joint article and find innovative ways to draft something beautiful.

### How does your collaboration work logistically? Who is involved from both sides?

**MW:** So, the three of us have never actually met in person; yes, not even myself and Isabelle, who became a close, personal friend over the years. Due to the pandemic and something about mice, men, and nicely laid plans, it just did not happen. Since we all live in different corners of the country, it was also just more convenient to collaborate remotely, rather than fit travel into our busy schedules or force an inaccessible public transport trip on my chronically ill body. We have collaborated through Microsoft Teams, as far as that program allowed. Juggling different institutional Microsoft accounts is A Hassle, but we managed.

### How important was the collaboration to the success of the paper? How important do you think collaboration is in general to research?

**BW:** This paper would have been impossible without lots of collaboration at an equal level, jointly critiquing each other heavily and then rewriting what we’d just written. There was also a lot of mutual respect for each other’s research from the start, which helped a lot to keep improving the article and take setbacks in our stride.

### What advice would you give other (data) scientists looking to establish collaborations

**BW:** Articles you write alone will be much more boring than article you write together.

**MW:** Hear, hear!

### What’s next for both of your teams? Can we expect more collaborations?

**BW:** Hopefully yes. Maranke no longer works at Utrecht University, although they are still finishing their PhD, which certainly poses challenges in terms of joint collaboration opportunities.

It’s sad that there often isn’t space in academia for some of the best people, often because academic institutions aren’t always as supportive as they should be. Maranke has a great job working as a consultant outside of academia, but I sometimes hope for a world in which they could still choose to work full time in academia.

### What is your advice for future data scientists?

**IF:** I see increasing calls that place all responsibility for safeguarding public and ethical values on data scientists. Frameworks like the ones we studied are an example of that; you’re simply expected to know how to implement all these vague principles other people have come up with. But you don’t work in a vacuum. You have demanding managers, illiterate users, shifting priorities, and dynamic environments to deal with. Reality is fuzzy, and a long shot from the ideal world that policy and frameworks are often based on. If you really want to achieve responsible data science, don’t go at it alone. Hold these others to account. Share your worries and your limitations. Give a little pushback when responsibilities are shifted to you. Although you have much discretion, you are not all powerful.
